# Occurrence of *Angiostrongylus cantonensis* in invasive snails in the French territories of America, French Guiana, Guadeloupe, and Martinique

**DOI:** 10.1371/journal.pntd.0014024

**Published:** 2026-03-03

**Authors:** Céline Dard, Dorothée Harrois, Loïc Epelboin, Magalie Pierre-Demar, Noémie Vireeye, Gélixa Gamiette, Régis Delannoye, Lydéric Aubert, Stéphanie Guyomard-Rabenirina, Séverine Ferdinand, Antoine Talarmin, Nicole Desbois-Nogard

**Affiliations:** 1 Team Host-Pathogen Interactions and Immunity to Infection, Institute for Advanced Biosciences (IAB), INSERM U1209 - CNRS UMR5309, Université Grenoble Alpes, Grenoble, France; 2 Laboratoire de Parasitologie-Mycologie, CHU Grenoble Alpes, Grenoble, France; 3 Laboratoire de biologie médicale, Centre Hospitalier de Basse-Terre, Basse-Terre, Guadeloupe; 4 Unité des Maladies Infectieuses et Tropicales, Centre Hospitalier Universitaire de Guyane, Cayenne, French Guiana; 5 Centre d’Investigation Clinique Inserm, UMR UA 17 Inserm Santé des Populations d’Amazonie, Centre Hospitalier Universitaire de Guyane, Cayenne, French Guiana; 6 Laboratoire Hospitalier Universitaire de Parasitologie & Mycologie, Centre Hospitalier Universitaire de Guyane, Cayenne, French Guiana; 7 Tropical Biomes and Immunophysiopathology, UMR, Université de Guyane, Cayenne, French Guiana; 8 Unit of Health and Environment, Institut Pasteur de Guadeloupe, Les Abymes, Guadeloupe; 9 Association Martinique Entomologie, Fort-de-France, Martinique; 10 Santé publique France, Gourbeyre, Guadeloupe; 11 Laboratoire de Parasitologie-Mycologie, Centre Hospitalier Universitaire de Martinique, Fort-de-France, Martinique; Oregon State University College of Veterinary Medicine, UNITED STATES OF AMERICA

## Abstract

**Background:**

*Angiostrongylus cantonensis* is the primary etiological agent of eosinophilic meningitis, transmitted through rats (definitive hosts) and molluscs (potential intermediate hosts). Human infection occurs accidentally through the ingestion of contaminated hosts. First reported in the French West Indies in 2002, cases of *A. cantonensis* infections are emerging due to the spread of its hosts, particularly the invasive African giant snail (*Lissachatina fulica*). This study aims to assess the prevalence of *A. cantonensis* in gastropods across Guadeloupe, Martinique, and French Guiana, providing insights into its transmission dynamics.

**Methodology/Principal Findings:**

Terrestrial gastropods were collected in 2017 from residential areas with prior human cases and other selected sites with no reported case. The gastropods’ species were identified by a malacologist and processed for DNA extraction. Molecular diagnosis of *A. cantonensis* was performed using quantitative PCR. Prevalence rates were analyzed by Chi-square and Kruskal-Wallis tests, while correlations between weight and level of *A. cantonensis* DNA detected were assessed via Spearman’s rank correlation. A total of 430 gastropods, representing nine species, were collected: 103 from Guadeloupe, 161 from Martinique, and 166 from French Guiana. The highest prevalence of *A. cantonensis* was observed in Guadeloupe (38.8%), followed by Martinique (27.6%) and French Guiana (15.7%). Sampling in Martinique included a wider diversity of gastropod species, whereas collections in French Guiana and Guadeloupe were limited to *Lissachatina* spp. In Martinique, eight species were identified, five of which were infected. The prevalence was positively correlated with weight in the primary intermediate host, *L. fulica*. Interestingly, *Lissachatina immaculata* in French Guiana was confirmed as a potential intermediate host for the first time.

**Conclusions/Significance:**

The study highlights *L. fulica* as the predominant host in Guadeloupe and Martinique, while *L. immaculata* plays a significant role in French Guiana. Gastropod diversity and prevalence varied across territories, influenced by collection methods and ecological factors. Despite high infection rates in gastropods, human cases remain relatively rare, likely due to limited human-gastropod interaction and local culinary practices. These findings underscore the need for further investigation into transmission dynamics and phylogenetic studies to inform public health strategies.

## Author summary

We studied the presence of the parasite *Angiostrongylus cantonensis*, also known as the rat lungworm, which can cause eosinophilic meningitis in humans. This parasite usually lives in rats, but humans can become accidental hosts if they eat raw or undercooked snails or other small infected animals. First reported in the French West Indies in 2002, cases of *A. cantonensis* infections are emerging due to the spread of its intermediate hosts, particularly the invasive African giant snail (*Lissachatina fulica*), but very little was known about how widespread it might be in this region. To answer this question, we collected snails in Guadeloupe, Martinique, and French Guiana and tested them for the parasite. We found that *L. fulica* was the main carrier in Guadeloupe and Martinique, while another species, *Lissachatina immaculata*, played an important role in French Guiana. Infection rates in snails were high, but human cases remain rare, likely because people in these regions do not often eat raw snails. Our work shows that different snail species contribute to the spread of the parasite in different geographical regions, and that further studies into the dynamics of transmission are therefore important for protecting human health.

## Introduction

The rat lungworm, *Angiostrongylus cantonensis* (Order: Strongylida, Superfamily: Metastrongylidae, Family: Angiostrongylidae, Genus: *Angiostrongylus*) (https://www.itis.gov/), is the primary causative agent of eosinophilic meningoencephalitis in humans and animals [1,2].

The life cycle of *A. cantonensis* involves rats as definitive hosts, where adult helminths reside in the pulmonary arteries and right heart [3], and molluscs as intermediate hosts, mainly the African giant snail *Lissachatina fulica* (Bowdich, 1822), where larval maturation occurs, resulting in third-stage larvae (L3) [4]. Paratenic hosts which ingest contaminated molluscs may also facilitate the passive transport of L3 larvae, though they do not ensure larval maturation. These hosts include terrestrial planarians, amphibians (e.g., toads, frogs), and reptiles (e.g., monitor lizards) [5–9]. Crustaceans (e.g., shrimps, mangrove and terrestrial crabs) have also been proposed as paratenic hosts, though several authors suggest they may function only as transport hosts [10].

Human infection occurs accidentally *via* the ingestion of L3 larvae from raw intermediate or paratenic hosts. Humans are considered to be dead-end hosts. The larvae mainly migrate in the vessels of the central nervous system (CNS), where they cause inflammation. The most common symptoms are severe headache and neurological manifestations including eosinophilic meningitis, encephalitis/encephalomyelitis, paresthesia, radiculitis, cranial nerve abnormalities, and ataxia [11]. Although most infections are benign and resolve without treatment, some cases can be fatal, particularly in young children, who are at highest risk [12]. *A. cantonensis* is currently the leading infectious cause of eosinophilic meningitis worldwide, though it remains relatively rare, with approximately 3,000 documented cases in the international literature [1,13].

*Angiostrongylus cantonensis* was first described in 1935 in Guangzhou (formerly Canton), China, within the pulmonary arteries of domestic rats [14]. The first human case was diagnosed in 1945 in Taiwan, where parasitic larvae were detected in the cerebrospinal fluid (CSF) of a patient with eosinophilic meningitis [15], followed by several epidemics of eosinophilic meningitis in the Pacific Islands [16,17]. It was not until 1961 that Alicata established the link between *A. cantonensis* and meningitis epidemics, naming the disease [18]. Human cases of nerve angiostrongyliasis have since been reported in Asia (Thailand, Malaysia, China) [19,20], the Pacific Islands [21], the Indian Ocean Islands [12], the Greater Antilles (Cuba, Puerto Rico, Jamaica) [22], and more recently in South America [23].

*A. cantonensis* is now widespread, particularly in tropical countries, due to the extensive distribution of intermediate hosts. The global spread of *A. cantonensis* is likely facilitated by international trade (air and sea) and climate change, which have enabled the dispersal of definitive and intermediate hosts, especially the giant African snails (*L. fulica*) [24].

The first human cases in the French West Indies were reported in Martinique in 2002 and in Guadeloupe in 2013, with *A. cantonensis* also documented on the Guiana Shield in 2017. Since these initial reports, several cases have been described in Martinique and Guadeloupe [25–27]. This emergence of *A. cantonensis* in the French West Indies is likely linked to the recent introduction of *L. fulica* in this region, which was first noted in the Caribbean, particularly in the French islands in the 1980s. However, little is known about the potential intermediate hosts of *A. cantonensis* in these regions.

The objective of this study was to evaluate the prevalence of *A. cantonensis* in the most common host mollusc species to better understand the parasite’s life cycle and transmission dynamics in the French overseas territories of Guadeloupe, Martinique and French Guiana.

## Materials and methods

### Settings

**Fig 1:**
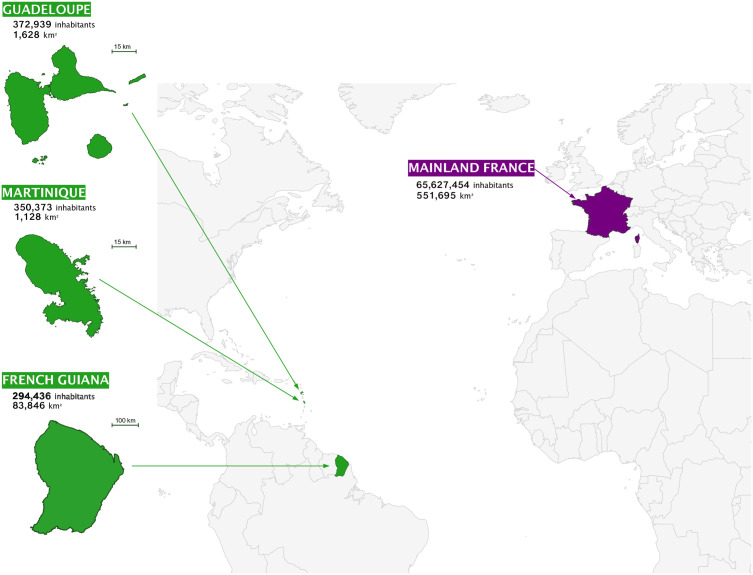
Location of geographical areas. The regions studied are shown in green. Administrative boundaries for Guadeloupe, Martinique, and French Guiana were taken from open French GeoJSON datasets (notably the france-geojson repository (https://github.com/gregoiredavid/france-geojson), which provides commune, department, and overseas DROM-level geometries derived from IGN Admin Express/ INSEE (https://github.com/gregoiredavid/france-geojson/blob/master/communes-avec-outre-mer.geojson) and is distributed under the Open Licence Etalab 2.0 https://www.data.gouv.fr/pages/legal/licences/etalab-2.0). Additional French administrative contours were sourced from the data.gouv.fr “Contours administratifs” dataset https://www.data.gouv.fr/datasets/contours-administratifs/), released under the Open Database Licence ODbL v1.0 (https://opendatacommons.org/licenses/odbl/1-0/). The global basemap for the world view was obtained from the public-domain Natural Earth Admin 0 countries dataset (https://www.naturalearthdata.com/downloads/110m-cultural-vectors/110m-admin-0-countries/; terms of use: https://www.naturalearthdata.com/about/terms-of-use/). All layers were then plotted as vector polygons and exported as editable.svg,.pdf, and.eps format for final layout in Affinity Designer. Conception: Bénédicte Sauvage, Bcom, French Guiana, Dr. Loic Epelboin, Dr. Céline Dard, and Dr. Christopher Swale.

French Guiana, Guadeloupe, and Martinique are three French overseas territories located in the Americas (Fig 1). Martinique and Guadeloupe are islands situated in the Lesser Antilles of the Caribbean. Guadeloupe is an archipelago of 1,628 km^2^, composed mainly of two large islands - Grande-Terre and Basse-Terre - separated by a narrow strait, along with several smaller islands including Marie-Galante, La Désirade, and Les Saintes. Located further south, Martinique spans an area of 1,128 km^2^. Both islands exhibit rugged terrain due to their volcanic origin. The climate is tropical, characterized by two main seasons: a wet season from July to November, marked by frequent rainfall, and a dry season from January to mid-April, with predominantly sunny and dry weather. In contrast, French Guiana is located on the northeast coast of South America, bordered by Brazil to the east and south, and Surinam to the west. It covers a vast area of 83,846 km^2^, over 90% of which is covered by Amazonian rainforest. The region has a humid equatorial climate, governed by the seasonal movements of the Inter Tropical Convergence Zone. The annual climate cycle includes a short rainy season from December to mid-February, followed by a transitional dry spell known as the “short dry season in March” from mid-February of late March. The long rainy season then extends from April to August, followed by a dry season from September to November.

### Sampling of gastropods

Terrestrial gastropods were primarily collected in residential areas of Guadeloupe and Martinique where human cases of *A. cantonensis* infection had been previously confirmed [25,26]. Additional sampling was conducted in other communes of Guadeloupe, Martinique and across French Guiana, where a network of volunteers – including scientists, naturalists, and medical professionals – contributed to specimen collection. The snails were collected in July 2017 in Martinique, January to March and June 2017 in Guadeloupe and June and July 2017 in French Guiana.

**Fig 2:**
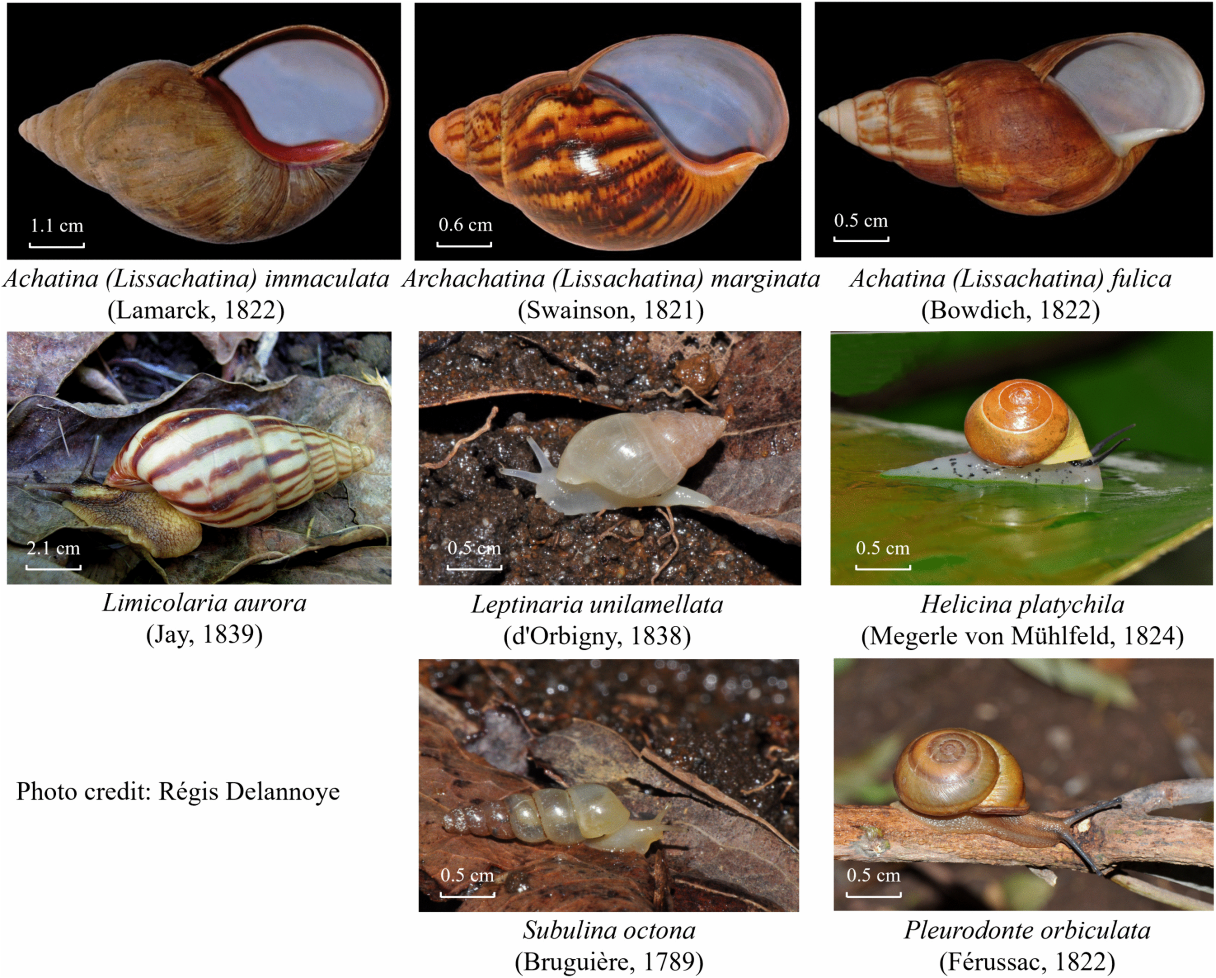
Morphological and visual characteristics of *Lissachatina* shells. Photo Credit: Régis Lannoye, Conception with Séverine Ferdinand.

All gastropods were weighted and identified to species level by morphology using standard diagnostic characters (shell morphology, aperture shape, columellar and spire traits, and soft-body features when available), following the taxonomic keys and regional inventories, particularly those of Delannoye et al. 2015 for Guadeloupe and Martinique [1], and of Massemin et al. 2009 for French Guiana [2]. Species identifications were confirmed by the malacologist in our team (Régis Delannoye) and illustrated in Fig 2. We chose to follow Delannoye et al. taxonomy for consistency with regional faunal works, but we acknowledge that the elevation of *Lissachatina* to genus level is not currently supported by robust phylogenetic evidence.

### Molecular diagnosis of *A. cantonensis*

For practical reasons, snails collected in Martinique and French Guiana were dissected in on-site laboratories of parasitology; the samples were then frozen and sent to the Institut Pasteur de Guadeloupe for DNA extraction. Snails collected in Guadeloupe were frozen whole at -20°C and thawed prior to dissection at the Institut Pasteur.

All specimens were dissected as follows: the shell was first broken and removed, after which two different anatomical parts were removed using forceps and a scalpel for DNA extraction: the mantle (soft tissue beneath the shell) for snails from Guadeloupe, and the pallial bulge for snails from Martinique and French Guiana. The choice of tissue samples evolved during the study for practical reasons and because of an improved understanding of the optimal anatomical sites for detecting *A. cantonensis*. Initially, it was found that the pallial bulge was easier for non-specialists to dissect consistently. As the study progressed, we observed that the literature provides relevant evidence for higher concentrations of *A. cantonensis* larvae in the mantle compared to other snail tissues and recommends including mantle tissue in order to maximise detection sensitivity [28]. Consequently, for the frozen whole snail specimens from Guadeloupe, which were, we selected the yet available mantle tissue to maximise the sensitivity of *A. cantonensis* detection. Total DNA was extracted from each dissected tissue individually using the cetyltrimethylammonium bromide (CTAB) method. Detection of *A. cantonensis* was performed by amplification of the internal transcribed spacer 1 (ITS1), with specific primers AcanITS1F1 (5′-TTCATGGATGGCGAACTGATAG-3′) and AcanITS1R1 (5′-GCGCCCATTGAAACATTATACTT-3′) used for real-time PCR, as described previously [29]. Ct values reflect the DNA content of the analyzed tissue subsample, not the entire gastropod.

### Statistical analyses

The infection rate of gastropods with *A. cantonensis* was estimated by calculating the prevalence percentage (%) along with its 95% confidence interval (CI95%). Non-parametric statistical tests (Chi2 or Kruskal-Wallis test, as appropriate) were employed at the 5% significance level to compare the prevalence among different groups of gastropods based on department, municipality, species collected or weight of gastropods. The Spearman rank correlation test was used to assess the relationship between weight of snails and Ct values reflecting level of *A. cantonensis* DNA detected, as quantitative variables. Results are presented as medians with interquartile ranges for quantitative variables, and as frequencies and percentages for qualitative variables. Statistical analyses were performed using STATA 11.0 and GraphPad Prism version 6.04 for Windows.

## Results

A total of 430 gastropods of nine different species were collected between January and July 2017, in Guadeloupe (n = 103), in Martinique (n = 161), and in French Guiana (n = 166). In Guadeloupe, 103 gastropods of *L. fulica* species were collected from nine sites across seven among 32 communes, with numbers ranging from 2 to 49 per commune. No other species were found in Guadeloupe during our collections. In French Guiana, 166 gastropods of *Lissachatina immaculata* (Lamarck, 1822) were collected from 15 sites in six among 22 communes, with numbers ranging from 5 to 55 per commune. In Martinique, 161 gastropods representing eight species (*L. fulica*, *Archachatina marginata* (Swainson, 1821), *Limicolaria aurora* (Jay, 1839), *Semperula wallacei* (Robinson, 2012), *Helicina platychila* (Megerle von Mühlfeld, 1824), *Leptinaria unilamellata* (d’Orbigny, 1838), *Subulina octona* (Bruguière, 1789), and *Pleurodonte orbiculata* (Férussac, 1822)) were collected from eight sites in five among 34 communes, with numbers ranging from 1 to 105 gastropods per commune.

### Occurrence and geographical distribution of gastropods infected with *A. cantonensis*

**Fig 3:**
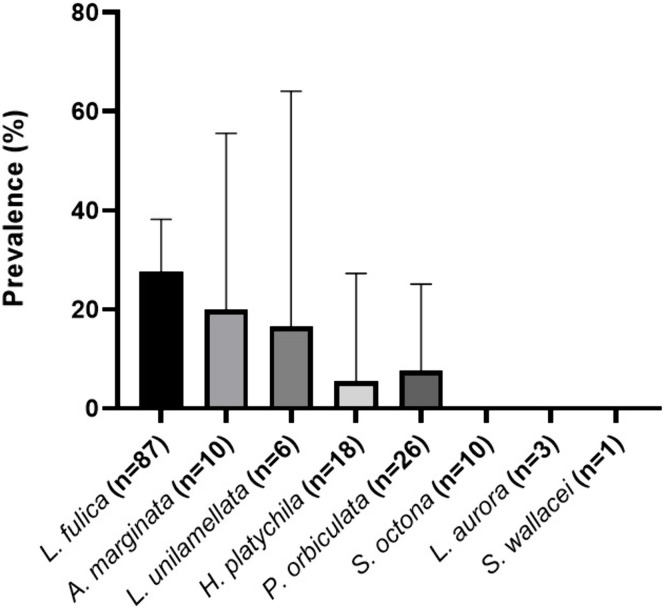
Prevalence of *A. cantonensis* infection in gastropod species collected from various communes in Martinique.

Among the eight species of gastropod found in Martinique, three (*S. octona, L. aurora, S. wallacei)* were not infected with *A. cantonensis*, while five (*L. fulica, A. marginata, L. unilamellata, P. orbiculata, and H. platychila)* were infected. Prevalence rates varied from 5.6% (95% CI [0.1; 27.3]) in *H. platychila* to 27.6% (95% CI [18.5; 38.2]) in *L. fulica* (Fig 3). Significant differences in prevalence were observed between *L. fulica* and *P. orbiculata* (27.6% vs 7.7%) and between *L. fulica* and *H. platychila* (27.6% vs 5.6%) (p < 0.05). These results suggest that *L. fulica* serves as a more frequent host for *A. cantonensis* compared to *P. orbiculata* or *P. platychila*. No difference was observed between *L. fulica* and *A. marginata* and *L. unilamellata* (Fig 3).

**Fig 4:**
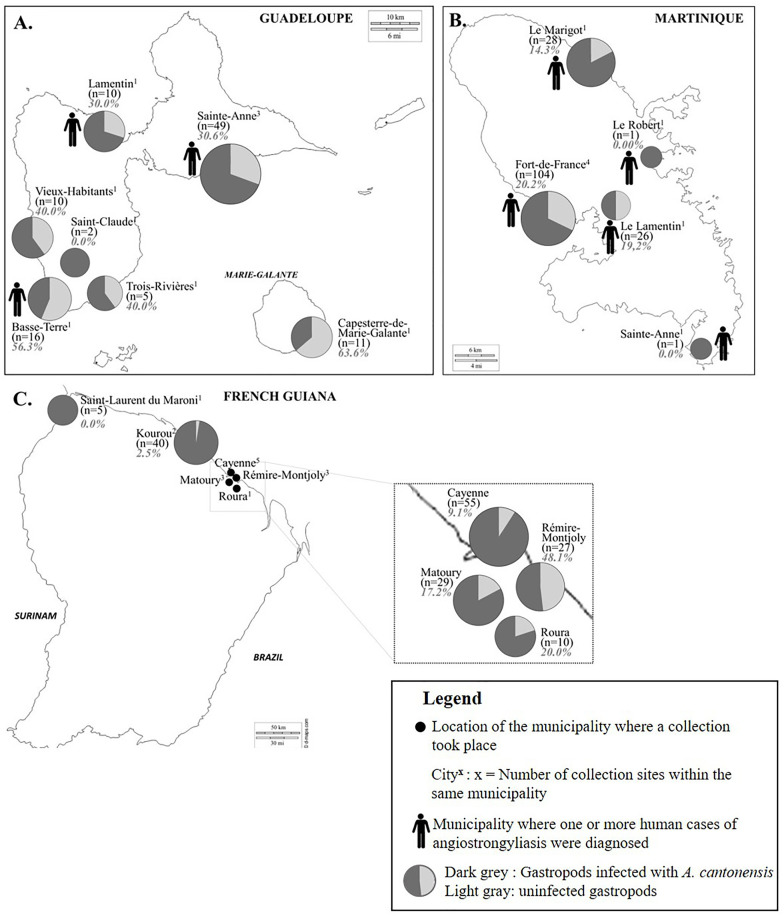
Geographical distribution of *A. cantonensis* in gastropods collected from communes in Guadeloupe (A), Martinique (B), and French Guiana (C). Superscript numbers following the city names indicate the number of collection sites within each commune. Human icons represent communes where one or more human cases of angiostrongyliasis eosinophilic meningitis were diagnosed prior to the environmental survey. In the pie charts, light gray sections denote the proportion of gastropods infected with *A. cantonensis*, while dark gray sections represent uninfected gastropods. Corresponding prevalence rates are indicated in gray and expressed as percentages (%). Base-layer map showing country borders was obtained from d-maps: Guadeloupe (https://d-maps.com/carte.php?num_car=2849&lang=fr), Martinique (https://d-maps.com/carte.php?num_car=2941&lang=fr), and French Guiana (https://d-maps.com/carte.php?num_car=2859&lang=fr). Maps are used in accordance with the d-maps terms of use (https://d-maps.com/conditions.php?lang=en). The icons, pie charts and clip art within the figure panels were drawn by hand.

The prevalence of *A. cantonensis* infection among *Lissachatina* species (*L. fulica* in Guadeloupe and Martinique, *L. immaculata* in French Guiana) were significantly different (Chi^2^, p = 0.0014). The highest prevalence was observed in Guadeloupe (38.8%, 40/103), followed by Martinique (18.6%, 30/161), and French Guiana (15.7%, 26/166). The prevalence rates for each commune across the three regions are detailed in Fig 4. In both Guadeloupe and Martinique, the prevalence of *A. cantonensis* in gastropods did not differ significantly between communes (p > 0.05). However, a significant “decreasing gradient” in infection prevalence was observed from northeastern French Guiana (Rémire-Montjoly > Roura > Matoury > Cayenne) to northwestern French Guiana (Kourou > Saint-Laurent du Maroni) (p < 0.0013) (Fig 4C).

### Profiles of *L. fulica* infected with *A. cantonensis*

Specimens of *L. fulica* collected in Guadeloupe and Martinique (n = 189) were categorized into three weight classes: low weight (< 10 g), medium weight (10–20 g), and high weight (> 20 g), to test the hypothesis that the prevalence of *A. cantonensis* infection varies with the weight of *L. fulica*. This species was selected for evaluation as it is the primary intermediate host of *A. cantonensis* and represents the most abundant gastropod collected in this study, accounting for 44.0% (189/430) of the samples. Weight data for *L. fulica* were unavailable for 18 specimens.

**Fig 5:**
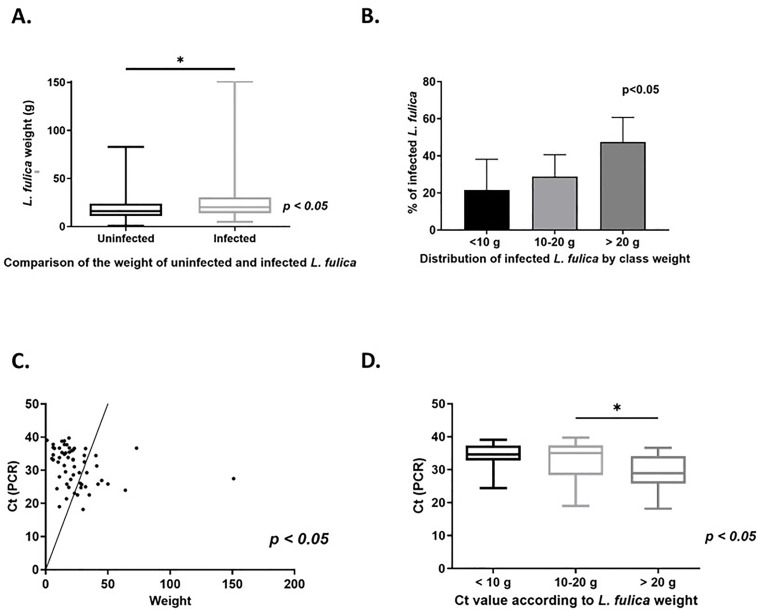
Relationship between the weight of *Lissachatina fulica*, the prevalence of *A. cantonensis* infection, and level of *A. cantonensis* DNA detected. **(A)** Comparison of the weight of infected (*n* = 57) and uninfected (*n* = 103) *L. fulica*. **(B)** Comparison of the prevalence of *A. cantonensis* infection across three weight categories: < 10 g (*n* = 9), 10-20 g (*n* = 20), and >20 g (*n* = 28). **(C)** Assessment of the correlation between weight and level of *A. cantonensis* DNA detected (Ct) in *L. fulica*. **(D)** Comparison of level of *A. cantonensis* DNA detected based on the three weight categories among infected *L. fulica*. Quantitative variables are summarized as median and interquartile range [iqr]. Qualitative variables are presented as numbers and percentages. Intergroup differences were assessed using the Mann-Whitney test or chi-square test, as appropriate.

The prevalence of *A. cantonensis* infection increased significantly with weight category, from 22.0% in the < 10 g group to 47.5% in the > 20 g group (p = 0.0150) (Fig 5B). Infected *L. fulica* (n = 58) had a significantly higher median weight than uninfected individuals (n = 113), with weights of 19.6 g [9.0-22.0] vs 15.0 g [13.2-30.0], respectively, p < 0.0060) (Fig 5A). The cycle threshold (Ct) obtained from PCR analyses (inversely proportional to level of *A. cantonensis* DNA detected) negatively correlated with the weight of *L. fulica* (Spearman’s rho = -0.40, p < 0.0018) (Fig 5C). There was a significant decrease in Ct from 35.6 to 28.6 with increasing weight category (n = 58, p = 0.0083) (Fig 5D). This result suggests a positive relationship between *L. fulica* weight and level of *A. cantonensis* DNA detected.

In French Guiana, infected *L. immaculata* had a significantly lower median weight than uninfected *L. immaculata* (22.5 g [8.0-43.0] vs 40.0 g [22.0-59.5] respectively, p = 0.0027). However, the Ct obtained from PCR analyses (inversely proportional to level of *A. cantonensis* DNA detected) showed no significant correlation with the weight of *L. immaculata* (p = 0.1441).

## Discussion

Due to the recent emergence of *A. cantonensis* in the Americas, and especially in the French overseas territories of the Americas, it was essential to identify the potential intermediate hosts of this parasite in these regions and to assess the prevalence of infected gastropods to evaluate the risk to humans. Our study shows that the prevalence of *A. cantonensis* in gastropods is high in all three territories. In Guadeloupe and Martinique, the species *L. fulica* is largely predominant and appears to be the main terrestrial molluscan intermediate host, although other gastropods species have been found to be positive. The level of *A. cantonensis* DNA detected in *L. fulica tissues* was correlated with its weight, as previously demonstrated in similar studies on *L. fulica* [30,31] but also contradicted in others [32]. This finding is likely related to an increasing risk for gastropods of becoming infected with age (and thus indirectly weight), as well as increased ingestion of food and consequently L1 environmental larvae.

A possible explanation for the significantly lower weight of *L. immaculata* infected with *A. cantonensis* in French Guiana is that rat feces provide a richer source of calcium for *L. immaculata* than is available in their natural environment. Indeed, snails need calcium for shell growth, and rat feces can be a source of this essential nutrient, especially in environments such as French Guiana where calcium availability in the soil is low. This behavior is less common in adult snails because their need for calcium decreases once their shells are fully formed.

In the present study, the diversity of gastropod species is higher in Martinique than in Guadeloupe and French Guiana, with 9 species tested in Martinique compared to only one in the two other territories. This does not reflect the respective biodiversity of the territories, since there are an estimated 76 species of terrestrial and freshwater molluscs in Martinique [33,34], 96 species in Guadeloupe [35] and 96 in French Guiana. [36]. In French Guiana and Guadeloupe, the collection of snails was specifically focused on African giant snails by non-malacologists, whereas in Martinique, collectors were accompanied by a malacologist, which led to the inclusion of other species. Among the species collected, *Helicina platychila* and *Pleurodonte orbiculata* are considered native to Martinique, whereas *L. fulica*, *Archachatina marginata*, *Limicolaria aurora*, *Semperula wallacei*, *Leptinaria unilamellata*, and *Subulina octona* are introduced exotic species. Interestingly, *H. platychila* that had never been found infected by *A. cantonensis,* has now been identified as a potential intermediate host in Martinique. However, its prevalence of infection was significantly lower than that of *L. fulica*, probably related to a reduced susceptibility to infection and their smaller size, thus a lower risk of being infected. Finally, the genus *Pleurodonte* had previously been found to carry the parasite without the exact species being determined [37], and our study showed that *Pleurodonte orbiculata* could be infected by *A. cantonensis*. *Archachatina marginata* has already been reported as a potential carrier of *A. cantonensis*, but the literature is scarce [38,39]. Caution is warranted when interpreting the role of newly reported gastropod species capable of carrying *A. cantonensis*, as detection of parasite DNA may reflect transient carriage rather than true intermediate host status. Their capacity to support larval development has yet to be demonstrated.

The species *L. immaculata*, introduced in 1996 in French Guiana, is well established in urbanized areas of French Guiana, particularly in Cayenne, Matoury, Rémire-Montjoly and more recently Saint-Laurent du Maroni [40]. The report of the Regional Directorate of the Environment (DIREN) in 2010 classified it as a species “in latency” to be monitored and whose invasive potential had not yet been expressed. Moreover, this report already warned about the potential future involvement of this species in the emergence of angiostrongyliasis due to *A. cantonensis* [40]. Our study proved for the first time, to our knowledge, that the species *L. immaculata* can indeed be a vector of *A. cantonensis* and may contribute to human infections. Even if cases of nervous angiostrongyliasis may be underreported in French Guiana, the very particular presentation (neurological symptoms and significant hyper eosinophilia in the blood and cerebrospinal fluid), combined with the presence of experienced infectious diseases specialists on the territory, suggest that underdiagnosis is likely limited. Thus, there is a contrast between the ubiquity of *L. immaculata* in the urban coastline areas, the relatively high prevalence of the parasite in these gastropods, and the low number of reported cases, with only one case describe in the literature [27].

Several explanations can be proposed: limited spontaneous contact between humans and snails; snails are quite systematically destroyed by the population, especially among the more educated who are aware that they carry disease; limited contact with the most vulnerable individuals, as seen in Mayotte [12]; snails are rarely eaten here; the agricultural areas in French Guiana are far from urban areas where *L. immaculata* is found, reducing the risk of plants contamination, etc. In addition, the semi-limpet *Parmarion martensis*, recorded for the first time in French Guiana in 2006 in Cayenne and Saint-Jean du Maroni [41], and classified among the 13 invasive exotic species in French Guiana by the Regional Scientific Council of Natural Heritage (CSRPN) [40], also requires monitoring; it was observed in Martinique in 2024. Indeed, it is one of the major potential intermediate hosts of *A. cantonensis* in the Pacific and Southeast Asia, and has caused series of cases of nerve angiostrongyliasis in Hawaii in recent years [42]. This species was not found during our environmental collections, likely because they occurred during a relatively dry period. New environmental collections planned during the rainy season will help evaluate its role in the transmission of *A. cantonensis* in French Guiana.

The prevalence of *A. cantonensis* infection of *Lissachatina* sp. is lower in French Guiana than in Guadeloupe and Martinique. This difference could be explained by a lower susceptibility of *L. immaculata* to infection, a later introduction of *Lissachatina* sp. and thus of the parasite into the Guianese territory, associated with a lower parasite multiplication, although numerous factors remain unresolved. Furthermore, the higher prevalence in Guadeloupe compared to Martinique and French Guiana could be partly due to the fact that the mantle of the mollusc probably harbours a higher concentration of larvae than the pallial bulge, which was collected from molluscs in Martinique and French Guiana [43]. Finally, these differences could also be related to epidemiological differences in terms of definitive hosts. *Rattus rattus* is mainly found in urban areas in French Guiana and more in arboreal areas in the West Indies.

The emergence of human angiostrongyliasis cannot be dissociated from that of its definitive and intermediate hosts, notably rats and the gastropod *L. fulica*, whose recent invasive multiplication must be considered in epidemiological models. *L. fulica* appears to have been introduced to Guadeloupe and Martinique in the early 1990s [44]. The diagnosis of the first cases of nerve angiostrongyliasis in Guadeloupe and Martinique, in 2013 and 2002, respectively, is thus perfectly consistent with the recent introduction of *L. fulica* in the Lesser Antilles and the fact that it was found to be the main intermediate host in our study. In French Guiana, the first case of *A. cantonensis* was recorded in 2019, 23 years after the introduction of *L. immaculata* which is found to be the main potential intermediate host in this area.

## Conclusion

This study demonstrated that the main potential intermediate hosts of *A. cantonensis* are *L. fulica* in Guadeloupe and Martinique and *L. immaculata* in French Guiana. This explains why the first cases of nervous angiostrongyliasis were reported only after the introduction of these gastropods on these territories. However, despite this recent introduction, the prevalence of *A. cantonensis* in these gastropods is quite important and represents risk of contamination for the population. Fortunately, the culinary habits of the local population do not represent a risk factor, and the human cases detected were often children who had played with gastropods. Differences in sampling strategies and tissue selection are limitations of this study that may affect the observed distribution and detection of *A. cantonensis* across regions. However, all these results allowed us to alert the Regional Health Authorities, who are currently working on informing the public and medical community as well as implementing preventive strategies. Many aspects of *A. cantonensis* require further exploration in our territories, including its prevalence in intermediate, paratenic, and definitive hosts—some of which may yet need to be discovered and better understood—as well as the initiation or continuation of phylogenetic studies.
